# 11.7T MR Imaging Revealed Dilatation of Virchow–Robin Spaces within Hippocampus in Maternally Lipopolysaccharide-exposed Rats

**DOI:** 10.2463/mrms.mp.2015-0090

**Published:** 2016-05-06

**Authors:** Yasuhiro Ooi, Chizuko Inui-Yamamoto, Yoshichika Yoshioka, Akitoshi Seiyama, Junji Seki

**Affiliations:** 1 Division of Pathogenesis and Control of Oral Disease, Graduate School of Dentistry, Osaka University, Suita, Osaka 565-0871, Japan; 2 CREST, Japan Science and Technology Agency (JST); 3 Center for Information and Neural Networks (CiNet), National Institute of Information and Communications Technology (NICT) and Osaka University; 4 Biofunctional Imaging Laboratory, Immunology Frontier Research Center (IFRec), Osaka University; 5 Division of Medical Devices for Diagnoses, Faculty of Human Health Sciences, Graduate School of Medicine, Kyoto University; 6 Department of Biomedical Engineering, National Cerebral & Cardiovascular Center Research Institute

**Keywords:** dilated Virchow–Robin space, maternal infection, dentate gyrus, hippocampal damage, ultra high-field T_2_-weighted MR imaging

## Abstract

**Purpose:**

11.7 Tesla MRI was examined to detect Virchow–Robin spaces (VRSs) smaller than 100 μm in the rat brain. The effects of maternal exposure to lipopolysaccharide (LPS) were evaluated on basis of the number of dilated VRSs in the offspring rat brain.

**Methods:**

T_2_-weighted MRI with an in-plane resolution up to 78 μm (repetition time = 5000 ms, echo time = 35 ms, slice thickness = 250 μm, imaging plane, coronal) was applied to identify VRSs. The dilated VRSs were counted in the rat brain at 5 and 10 weeks of age. The dams of half the number in each group were treated with LPS during pregnancy, and the remaining half was employed as control. LPS injection in gestation period was used to simulate maternal infections, the method of which was widely accepted as a rat model inducing neuropsychiatric disorders in the offspring. Effects of LPS exposure on the offspring rat brain were statistically investigated.

**Results:**

VRSs as small as 78 μm were successfully detected by the ultra high-field MRI. All dilated VRSs were observed within lacunosum molecular layer of hippocampus, and molecular and granular layers of dentate gyrus around hippocampal fissure. In juvenile rats (5 weeks of age), the number of dilated VRSs was significantly increased in the prenatal LPS exposed rat brain (12.9 ± 2.4, n = 7) than in the control (5.3 ± 1.5, n = 7, *P* < 0.05), while in young adult rats (10 weeks of age), there was no significant difference in the number between the prenatal LPS exposed rat brain (3.6 ± 0.9, n = 5) and the control (2.6 ± 0.4, n = 5).

**Conclusion:**

The results of the present study suggested that maternal infection might cause dilatation of VRSs through neural damages especially in the dentate gyrus of the offspring rats. Thus, ultra high-field MRI can offer a promising diagnostic tool capable of determining the location of neonatal brain damage caused by maternal infections.

## Introduction

Virchow–Robin spaces (VRSs) are perivascular spaces of small blood vessels that penetrate the brain parenchyma. The VRSs can be detected by magnetic resonance imaging (MRI) only when they dilate larger than 1 mm in diameter in human brains. T_2_-weighted MRI is generally used to visualize VRS in the recent clinical studies^1^ because it is sensitive for detecting brain pathology with inflammation.^2^ In this study, we attempted to detect VRS smaller than 1 mm with a high in-plane resolution up to 78 μm using an ultra high-field T_2_-weighted MRI. With regard to their appearance on MRI, MR signal intensity of VRSs was as high as cerebrospinal fluid (CSF), since they contain interstitial fluid, and dilated VRSs were observed in round or oval shape with a smooth margin. Furthermore, it has been known that dilated VRSs in the brain tend to increase both in volume and in number with aging,^3^ and commonly appear bilaterally.^3^ Since recent studies have suggested that VRS provides a place of immune response,^4^ and associates with autism (ASD)^5^ and neurodegenerative diseases such as Parkinson’s disease (PD),^6^ Alzheimer’s disease (AD),^7^ and multiple sclerosis (MS),^3^ then VRSs were noted as an MRI marker of inflammatory activity and atrophy in the brain.^3,6,7^

It has been reported in several studies that maternal infection brings about brain damages in the offspring, although their detailed etiology remains unclear. Fever, placental damages, and pro-inflammatory cytokine induced by maternal infections were considered to affect fetal brain development. In rodent experiments, maternal periodontal infection has been demonstrated to result in placental damages to increase interferon γ within the fetal brain and to produce ultrastructural injury to the brain (demyelination in white matter),^8^ and maternal viral infections have been reported to lead to brain atrophy.^9^ Furthermore in human, there have been several reports about maternal infection leading to severe brain malformations, such as microcephaly,^10^ hydranencephaly,^11^ and encephalomyelitis.^12^ Damages in the offspring brain caused by maternal infection were possibly involved in impairment of the brain functions, such as behavior, learning, cognition, communication, and socialization, and may lead to attention deficit hyperactivity disorder,^13^ mental retardation,^14^ and neuropsychiatric disorders such as schizophrenia (SCZ) and ASD.^15^

Lipopolysaccharide (LPS), a key pro-inflammatory component of gram-negative bacteria, is known to activate immune system. Maternal exposure to LPS has been used as an animal model of maternal infections in many studies.^3^ Beloosesky et al.^16^ have recently demonstrated on T_2_-weighted MRI that the offspring of LPS-treated dams exhibited increased signal intensity in white and gray matters, which was suggestive of the site of inflammation. They did not mention any relation of the high-signal intensity with dilated VRSs.^3,6,7^

The purpose of this study was to evaluate the availability of 11.7 Tesla (T) MRI for detecting small VRSs in rat brain and to investigate the effects of maternal exposure to LPS on the offspring rat brain based on observation of dilated VRSs.

## Materials and Methods

### Animals and treatments

All experiments followed the “Principles of Laboratory Animal Care” (NIH publication No. 86-23, revised 1985), and the protocols were approved by the Osaka University Animal Care and Use Committee. In total, 24 male Wistar rats (CLEA Japan, Osaka, Japan) were used for experiments. A total of 14 rats were used for the observation at 5 weeks of age, and 10 rats were used for 10 weeks of age. The dams of half the number in each group were treated with LPS (LPS from *E. coli* o111:B4, Sigma Chemical, St. Louis, MO, USA) during pregnancy, and the remaining half was employed as control. We referred to a rat brain atlas to determine the location of the nervous tissue.^17^ LPS was intraperitoneally administered to pregnant dams at a dose of 100 μg/kg every second day from day 16 of pregnancy till delivery.

### MRI analysis

The rats were anesthetized by urethane (1.25 g/kg) at 5 or 10 weeks of age. MRI was performed on the rats with 11.7T MRI vertical wide bore scanner system (AVANCE 500 WB; Bruker BioSpin, Ettlingen, Germany) equipped with a volume RF coil (m2m Imaging Corp., Cleveland, OH, USA) having an inner diameter of 25 mm for transmission and reception. T_2_-weighted MRI that is highly sensitive to water was suitable for detecting brain pathology associated with inflammation.^2^ Therefore, we selected rapid acquisition with relaxation enhancement (RARE) sequence with T_2_-weighted parameters (T_2_-RARE) to demonstrate the effects of maternal LPS exposure during pregnancy on the offspring rat brain. Further to observe dilated VRS on the order of 100 μm, we used T_2_-RARE (repetition time (TR) = 5000 ms; echo time (TE) = 35 ms; and rare factor = 16) at a spatial resolution of 78 × 78 × 250 μm (field of view = 2.0 × 2.0 cm^2^; matrix size = 256 × 256; slice thickness = 0.25 mm; number of acquisitions = 16; and number of slices = 36). Total imaging time was about 42 min. The slice plane of MRI was adjusted so that it was perpendicular to the mid-sagittal plane and at 77 degrees anteriorly upward from the cranial base. In this study, cranial base, a plane consisting of presphenoids and sphenoid bones, was used as the horizontal reference plane in adult rats in place of the bregma and lamda point, since those structures cannot be observed by MRI.

**Fig 1.**
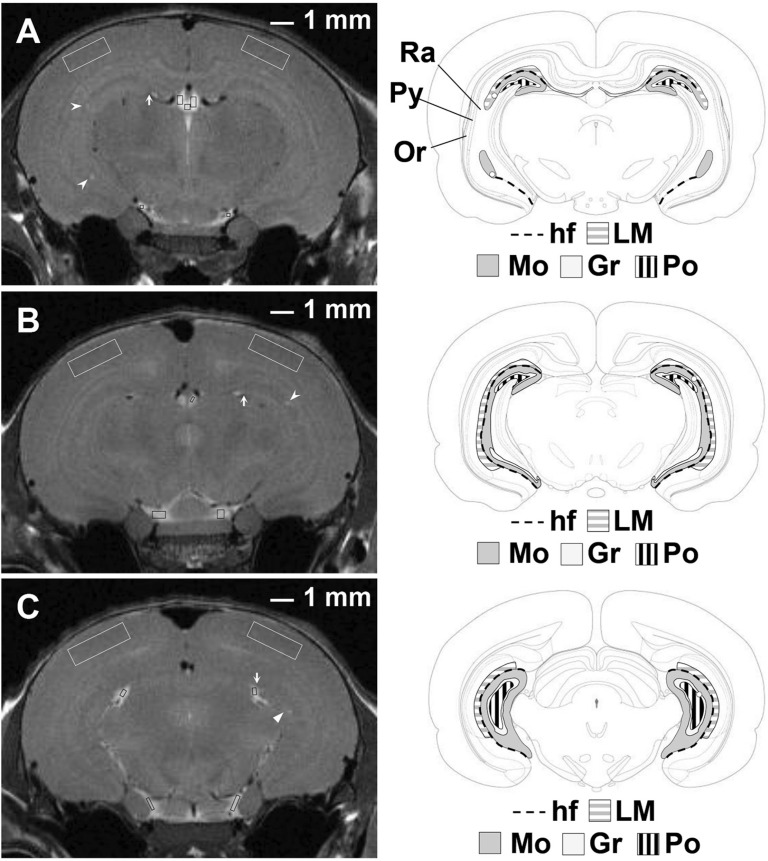
T_2_-weighted MR images obtained in a 5-week-old normal rat show bright spots within the region around hippocampal fissure (hf), which were considered dilated Virchow–Robin spaces (VRSs) (see text for determination of VRS). Left column: MR image of rat brain coronally sliced at (**A**) 4.90, (**B**) 5.40, and (**C**) 6.12 mm caudal from the bregma. Arrows indicate hf. Sharp arrowheads and triangular arrowheads point dilated VRSs within the low intensity area (coincident with lacunosum molecular layer of the hippocampus [LM], hf and molecular layer of the dentate gyrus [Mo]) and close to the medial side of low intensity area (coincident with granular layer of the dentate gyrus [Gr]), respectively. White and black rectangles indicate the regions of interest (ROI) for the mean intensity of cerebral cortex (CCm) and cerebrospinal fluid (CSFm), respectively. Right column: Rat brain anatomical structure^17^ corresponding to each MR image. Open circles indicate the anatomical positions corresponding to the arrowheads on MR images. The thick dotted line denotes hf. The horizontally hatched area represents LM, and the dark, medium, and light gray areas represent Mo, Gr, and polymorphic layer of the dentate gyrus (Po), respectively. hf, hippocampal fissure; LM, lacunosum molecular layer of the hippocampus; Mo, molecular layer of the dentate gyrus; Gr, granular layer of the dentate gyrus; Or, Oriens layer of the hippocampus; Py, pyramidal layer of the hippocampus; Ra, radiatum layer of the hippocampus; Po, polymorphic layer of the dentate gyrus.

**Fig 2.**
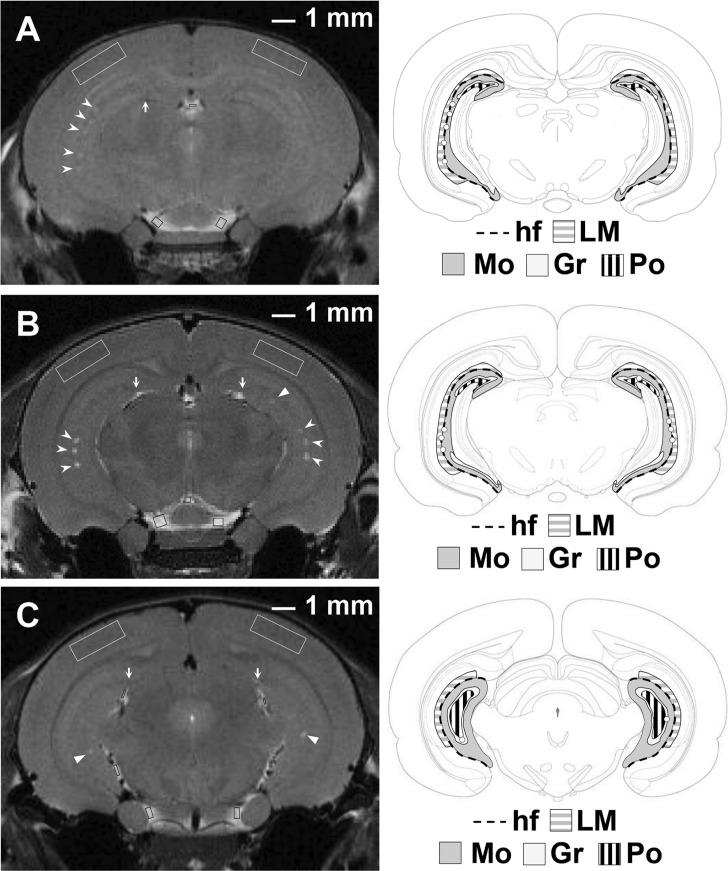
T_2_-weighted MR images obtained in a 5-week-old offspring rat of lipopolysaccharide (LPS)-treated dam show high intensity spots within the region around hippocampal fissure (hf), which were supposed dilated Virchow–Robin spaces (VRSs). Arrows indicate hf. Sharp arrowheads and triangular arrowheads point dilated VRS within low intensity area (coincident with lacunosum molecular layer of the hippocampus [LM], hf and molecular layer of the dentate gyrus [Mo]) and close to the medial side of low intensity area (coincident with granular layer of the dentate gyrus [Gr]), respectively. Left column: MR image of rat brain coronally sliced at (**A**) 5.28, (**B**) 5.40, and (**C**) 6.36 mm caudal from the bregma. White and black rectangles indicate the ROI for CCm and CSFm same as in Fig. 1. Right column: Rat brain anatomical structure corresponding to each MR image.^17^ Open circles indicate anatomical positions corresponding to the arrowheads on MR images. The thick dotted line denotes hf. The horizontally hatched area represents LM, and the dark, medium, and light gray areas represent Mo, Gr, and polymorphic layer of the dentate gyrus (Po), respectively.

VRS was characterized by round or oval shape with smooth margin and high-signal intensity on T_2_-weighted MR images as described in “Introduction.” Number of dilated VRSs were counted (by O.Y., 5 years of MRI training and/or experience) on 2–6 MR images covering the hippocampus and dentate gyrus. The signal intensity of dilated VRS was defined as the percent signal (Sr) being not <40, where Sr was calculated by the following equation:

(1) Sr(%)=(HISi−CCm)/(CSFm−CCm)×100

where HISi denotes the signal intensity of high intensity spot, CCm denotes the mean signal intensity of cerebral cortex, and CSFm denotes the mean signal intensity of cerebral spinal fluid in each MRI slice. The mean signal intensity of cerebral cortex and CSF was defined as the average intensity of 300–500 pixels and 100–200 pixels within the respective regions. Regions of interest (ROI) for CCm and CSFm were indicated by white and black rectangles, respectively (Figs. 1, 2).

## Statistical Analysis

**Fig 3.**
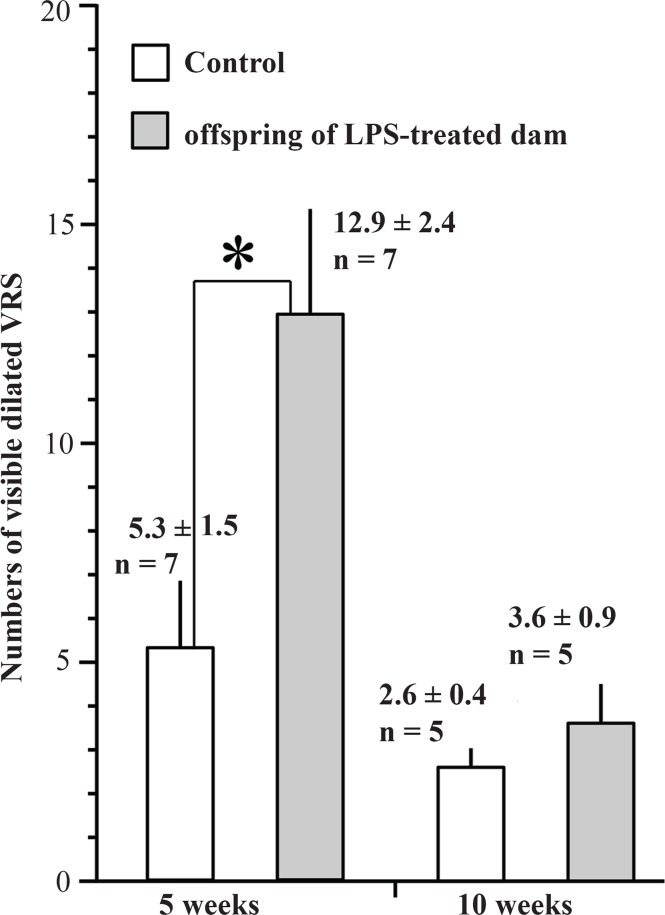
Average number of dilated Virchow–Robin spaces (VRSs) counted in the hippocampus and dentate gyrus of rat brain was compared between the offspring rats of lipopolysaccharide (LPS)-treated dams (gray bar) and the control (white bar). The number of dilated VRSs was significantly larger in 5-week-old offspring rats of LPS-treated dams than in the control rats at the same age, whereas the difference at 10 weeks of age was not statistically significant. The asterisk indicates the significant difference between the control rats and the offspring rats of LPS-treated dams (*P* < 0.05, two-way ANOVA, Student’s *t*-test). Data were expressed as mean ± standard error of the mean (SEM).

The results were expressed as arithmetic mean ± standard error of the mean (SEM) (Fig. 3). The two-way analysis of variance (ANOVA) was used to compare the number of dilated VRSs counted in the offspring rat brain at 5 and 10 weeks of age. The individual means were compared within the same age groups by the Student’s *t*-test (statistical significance was indicated by ^*^ for *P* < 0.05).

## Results

### MR images of 5-week-old normal rats

The left column of Fig. 1 shows the representative coronal sections of T_2_-weighted MRI for 5-week-old normal rat brain whose longitudinal position was (A) 4.9, (B) 5.4, and (C) 6.12 mm posterior to bregma. The right column shows the corresponding rat brain atlas.^17^ Sharp arrowheads and triangular arrowheads in the MR images of Fig. 1 indicate the presence of bright spots with high MR intensity. Figure 1a, b shows that the high-intensity spots were observed within the low-intensity area unilaterally, and Fig. 1c shows that there was one high-intensity spot close to the medial side of the low-intensity area. The diameter of these four spots ranges from 78 to 156 μm. The low-intensity area within the hippocampus in MRI was coincident with the lacunosum molecular layer of hippocampus (LM) and the molecular layer of dentate gyrus (Mo) shown in the rat brain atlas. The region close to the medial side of the low-intensity region in MR image was found to coincide with the granular layer of dentate gyrus (Gr) in the atlas. That is, the high-intensity spot (sharp arrowheads) in (A) and (B) locate within LM, hippocampal fissure (hf), or Mo, and the high-intensity spot (triangular arrowhead) in (C) locates within Gr. All the spots were appeared unilaterally. In the right atlas, open circles indicated the corresponding positions to the high-intensity spots on MR images.

### MR images of 5-week-old offspring rats of LPS-treated dams

The left column of Fig. 2 shows the representative coronal sections observed on T_2_-weighted MRI of 5-week-old offspring rat brain of LPS-treated dams. Their longitudinal position was (A) 5.28, (B) 5.4, and (C) 6.36 mm posterior to bregma. The right column shows the rat brain atlas^17^ corresponding to each MR image. Sharp arrowheads and triangular arrowheads in the MR images represent the same as in Fig. 1. Five high-intensity spots in (A) were observed in a line within the low-intensity area (LM, hf, or Mo) unilaterally and six high-intensity spots (sharp arrowheads) in (B) were within the low-intensity areas (LM, hf, or Mo) bilaterally. One high-intensity spot (triangular arrowhead) in (B) and two high-intensity spots (triangular arrowheads) in (C) appeared close to the medial side of the low-intensity area (Gr) unilaterally and bilaterally, respectively. The diameter of the 14 high-intensity spots in Fig. 2 ranges from 78 to 235 μm.

### Comparisons of the number of dilated VRSs between control rats and offspring rats of LPS-treated dams at 5 and 10 weeks of age

Figure 3 shows the average number of dilated VRSs counted in the hippocampus and dentate gyrus of the rat brain. We determined VRS according to its characteristic shape, size, and MR intensity described in “Introduction.” A two-way ANOVA showed that both LPS exposure (*P* < 0.025) and age (*P* < 0.005) significantly influence the average number of dilated VRSs. The number of dilated VRSs was significantly increased in the prenatal LPS-exposed rat brain (12.9 ± 2.4, n = 7) at 5 weeks of age than in the control (5.3 ± 1.5, n = 7), whereas, the difference at 10 weeks of age was not statistically significant. The asterisk indicates the significant difference between the control and prenatal LPS exposed rat (*P* < 0.05, as determined by the Student’s *t*-test). The error bars represent SEM.

## Discussion

The aim of this study was to investigate the effects of maternal infection on the offspring rat brain, using ultra high-field MRI with an in-plane resolution up to 78 μm and a slice thickness of 250 μm. The results of MRI scan showed the presence of small spots in round shape with high-signal intensity and smooth margin within the dentate gyrus and the hippocampus, especially in the offspring rats of LPS-treated dams. Cross-sectional shape of blood vessel was similar to dilated VRSs, however, their signal intensity on T_2_-weighted MRI was low due to flow void. Thus, these characteristic features of the high-intensity spots observed in this study most likely indicated that they were the dilated VRSs.

All the high-intensity spots observed in this study were located within the dentate gyrus and hippocampus. As shown Fig. 1, the dentate gyrus was divided into three layers: Mo, Gr, and polymorphic layer (Po), in the order of the distance to hf. Similarly, the hippocampus was divided into the following four layers: LM, radiatum layer (Ra), pyramidal cell layer (Py), and oriens layer (Or). The Gr and Py were cell layers, while the Mo and Or were relatively cell-free layers, and LM was next to Mo and resembles Mo in tissue structure.^18^ That is, the cell density of Mo and LM was lower than that of Gr, Py, and Ra, indicating that the signal intensity of the former is expected lower than the latter on T_2_-weighted MRI. Therefore, the low-intensity areas containing high intensity spots (sharp arrowheads in Figs. 1, 2) probably indicated Mo and LM. On the other hands, the triangular arrowheads located in the area inside Mo that coincides with Gr. Thus, all the expected VRSs were found in Mo, LM, and Gr. This was further supported by the observation in human that hippocampal sulcus remnant, which is known as dilated VRS,^19^ was frequently observed in the hippocampal sulcus (alias hippocampal fissure) by MRI.

Figure 3 indicates that maternal LPS exposure during pregnancy significantly increased the number of dilated VRSs within LM, Mo, and Gr, and the dilated VRSs have disappeared by 10 weeks of age. Since LPS activates innate immune cells such as dendritic cells, macrophages,^20^ and microglia^21^ to cause inflammation, maternal LPS exposure may cause chronic neuroinflammation in offspring rat brain at 5 weeks of age. In clinical researches, it has been reported that microglia were activated through epigenetic modulation in the brain of patients with neuroinflammation,^22^ PD,^23^ and AD,^24^ and that neuropsychiatric disorders were associated with low-grade inflammation in the brain.^25^ Intracortical injection of LPS in rats has been reported to induce microglial activation followed by inflammation, and chronic stress has been reported to increase susceptibility against brain inflammation.^21^ Thus, we expected that maternal LPS exposure induces the activation of microglia by epigenetic alteration. Since the epigenetic alteration was inherited even after cell division,^26^ activated microglia possibly survived in the offspring rat brain of LPS-treated dams at 10 weeks of age. If so, some psychological stress may stimulate the already activated microglia in the offspring rat brain to cause inflammation and dilatation of VRS. This estimate may account for the fact that the development of neurodegenerative diseases and psychiatric disorders were sensitive to stress, and that most patients with SCZ developed their disease between 16 and 25 years of age.^27^

In the maternal LPS exposure model, the offspring exhibits significant impairment in behaviors^28,29^ such as communication and socialization, and also shows a significant decrease in hippocampal neurogenesis within the dentate gyrus.^29^ Reduction in rat hippocampal neurogenesis has been reported to cause behavioral abnormality,^30^ the region of which has been known to contribute to cognitive functions such as memory and learning.^31^ These considerations suggested an association among maternal LPS exposure, abnormal behaviors appearing in neuropsychiatric disorders, and hippocampal damages^29,16^ in the offspring animals. In clinical studies, epidemiological survey suggested that maternal infection during gestation was a significant risk factor for neuropsychiatric disorders such as SCZ and ASD.^15^ In this context, the maternal LPS exposure was frequently used as a rat model inducing neuropsychiatric disorders in the offspring.

Neurogenesis, proliferation, and differentiation processes of neural stem cells in adult mammals were shown to persist only in the subgranular zone of the dentate gyrus (SGZ) and the subventricular zone.^32^ SGZ was a narrow layer between Gr and Po, and the neural stem cells migrate a short distance from SGZ to Gr in neurogenesis.^33^ Considering that, Gr was one of the regions where dilated VRSs were found in the present study, neurogenesis may also be obstructed by the same cause. Further, disappearance of dilated VRSs by the time of maturity of the offspring animal suggested that inflammation around dilated VRSs was reduced with aging. We speculated that the rise and fall of VRSs observed in the offspring rat may be a result of the following mechanism. Maternal LPS exposure stimulated type 1 helper T cells (Th1) rather than type 2 helper T cells (Th2) to result in imbalance between the cellular and humoral immune responses in offspring rats, where Th1 promotes cellular response and Th2 induces humoral responses. This situation was considered to continue up to 5 weeks of age and cause inflammation^34^ that inhibited the neurogenesis in dentate gyrus, and increased the number of dilated VRSs. The subsequent stress-free condition suppressed the immune responses,^21^ promoted the neurogenesis in dentate gyrus,^32^ and finally decreased the number of dilated VRSs to the control level until 10 weeks of age.

Microglia serve as resident innate immune cells that released pro-inflammatory molecules^35^ in the central nervous system, and microglia have been known to be linked to ASD,^36^ SCZ,^36^ and several inflammatory nerve diseases (alias neurodegenerative diseases) such as AD,^24^ MS,^37^ and PD.^23^ All these diseases but for SCZ were associated with dilated VRS.^3,5–7^ Microglia and perivascular macrophages have been reported to derive from primitive myeloid progenitors^38^ and monocytes,^39^ respectively. Therefore, it is important to monitor perivascular macrophages, monocytes, microglia, primitive myeloid progenitors, and neural stem cells in the vicinity of dilated VRSs *in vivo* so as to understand the mechanism in the development of SCZ, ASD, and the other neurodegenerative diseases. Up to the present, those cells except primitive myeloid progenitors: microglia,^40^ perivascular macrophages,^41^ monocytes,^42^ and neural stem cells^43^ have already been visualized using MRI by labeling them with superparamagnetic iron oxide (SPIO). Such a technique would allow investigators to get much important information on cellular responses in the vicinity of dilated VRSs in the brain of animal models with neuropsychiatric disorders.

Although little was known concerning the risk factors of dilated VRSs and their association with inflammation, dilated VRSs as well as inflammatory responses have been found in the brain of patients with ASD and neurodegenerative diseases.^3,5–7^ There were also several reports that those diseases^6^ and SCZ^44^ were associated with dysfunction of blood–brain barrier (BBB). Since the BBB dysfunction resulted in microvascular hyperpermeability, which was a major contributor of neuroinflammation, it was expected to be involved in the development of neuropsychiatric disorders and dilated VRS. Thus, it was very interesting to visualize functional permeability and dysfunction of BBB by MRI with the aid of manganese (Mn)^45^ and/or gadolinium diethylenetriaminepentaacetate (Gd-DTPA).^46^

In conclusion, an ultra high-field T_2_-weighted MRI revealed that maternal LPS exposure caused damages localized within the region around hippocampal fissure of juvenile offspring rats. The damages were observed as dilated VRSs on MR image at 5 weeks of age, and not at 10 weeks of age. It suggested that the maternal LPS exposure may cause inflammation within the region around hippocampal fissure of neonatal and embryonic offspring rats, and the damages attenuated with aging. Thus, the dilated VRSs can be a promising MRI marker of early stage of offspring brain damage originating from maternal infection with gram-negative bacteria with outer membrane containing LPS.
